# Structural Determination, Biological Function, and Molecular Modelling Studies of Sulfoaildenafil Adulterated in Herbal Dietary Supplement

**DOI:** 10.3390/molecules26040949

**Published:** 2021-02-11

**Authors:** Kanchanok Kodchakorn, Nawarat Viriyakhasem, Tunchanok Wongwichai, Prachya Kongtawelert

**Affiliations:** 1 Thailand Excellence Center for Tissue Engineering and Stem Cells, Department of Biochemistry, Faculty of Medicine, Chiang Mai University, Chiang Mai 50200, Thailand; kanchanok_k@cmu.ac.th (K.K.); tunchanok.wong@gmail.com (T.W.); 2 School of Traditional and Alternative Medicine, Chiang Rai Rajabhat University, Chiang Rai 57100, Thailand; nawarat.vir@crru.ac.th

**Keywords:** dietary supplement, NO releasing, gene expression, PDE5 inhibitors, sildenafil analogue, molecular dynamics simulation

## Abstract

Unapproved ingredients included in herbal medicines and dietary supplements have been detected as adulterated synthetic drugs used for erectile dysfunction. Extraction from a dietary supplement was performed to isolate the compounds by HPLC analysis. The structural characterization was confirmed using mass spectrometry (ESI-TOF/MS and LC-MS/MS), ^1^H NMR, and ^13^C NMR spectroscopy techniques. Results identified the thus-obtained compound to be sulfoaildenafil, a thioketone analogue of sildenafil. The biological activities of this active compound have been focused for the first time by the experimental point of view performance in vitro. The results revealed that sulfoaildenafil can affect the therapeutic level of nitric oxide through the upregulation of nitric oxide synthase and phosphodiesterase type 5 (PDE5) gene expressions. This bulk material, which displays structural similarity to sildenafil, was analyzed for the presence of a PDE5 inhibitor using a theoretical calculation. These unique features of the potential activity of PDE5 protein and its inhibitors, sildenafil and sulfoaildenafil, may play a key consideration for understanding the mode of actions and predicting the biological activities of PDE5 inhibitors.

## 1. Introduction

Herbal medicines or dietary supplements have been popularized and advertised as natural and safe for human consumption. Nevertheless, some herbal drugs are contaminated, including with synthetic chemical compounds used to adulterate their marketed products in order to enhance the effects of their products, in which it is claimed that they are able to help treat certain chronic ailments and diseases [1,2,3]. There have been numerous recent studies that have reported about herbal drugs for the treatment of erectile dysfunction to enhance male sexual performance [1,4,5,6]. Those published adulterants include the synthetic phosphodiesterase-5 (PDE5) inhibitors that do not only include FDA-approved drugs [7,8,9], but also their synthetic analogues, i.e., homosildenafil [10], thiohomosildenafil, thiosildenafil [11], and thiomethisosildenafil [12] through minor structural modifications. However, the presence of these drug analogues can cause some serious health risks and unexpected side-effects for patients, especially when their uses have not been clinically proven to be safe, resulting in unpredictable adverse effects.

Numerous analogues of synthetic PDE5 inhibitors, for example sildenafil, vardenafil, and tadalafil, have been studied in the cases (Figure S1, Supplementary Information) [7,8,9,13,14,15]. It has been observed that the development of hypertension has been associated with endothelial dysfunction characterized by oxidative stress and by decreasing endothelium-derived relaxing factors, such as the consumption of nitric oxide (NO) [16]. NO is synthesized through the activity of nitric oxide synthase (NOS) enzymes by expressing in the endothelium of arteries and the neuron cells [17]. Current reports have proposed that, in general, analogues of sildenafil display a variety of cellular functions, including muscle relaxation, anti-inflammation, and signal transduction [18], in addition to displaying beneficial effects of sexual endothelial dysfunction and pulmonary hypertension. According to this situation, when sexual stimulation causes a local release of NO, the synthetic inhibitory effects of PDE5 creates a retaining intracellular cyclic guanosine monophosphate (cGMP) levels, resulting in muscle relaxation and an inflow of blood into the corpus cavernosum penis.

Almost all herbal supplements were detected to contain adulterated products with sildenafil analogues, which can be obtained over the counter at regular drugstores. One of the most powerful techniques for structural determination of the isolated compounds in herbal extracts is NMR (^1^H- and ^13^C-NMR) spectroscopy [2,5]. Furthermore, these synthetic compounds have also been investigated and presented structural similarities to sildenafil by means of UV spectroscopy, liquid chromatography (LC), high-resolution mass spectroscopy (MS), and X-ray structure analysis [1,4,10,12,14]. However, in many cases, there is no information available regarding the potential toxicological or pharmacological effects on the public.

Here, we demonstrated that sulfoaildenafil, a thioketone analog of sildenafil, has been detected as an adulterant in herbal aphrodisiacs. The effects of the isolated compound have been focused for the first time from both of the structural characteristics and the experimental point of view performance in vitro. The present study was designed to determine its effects on the human umbilical vein endothelial EA.hy926 cells, focusing the role of toxicity, NO-releasing levels, and the regulation gene expression of NO synthesis and PDE5 inhibitory effect. Finally, this bulk material, which displays structural similarity to sildenafil, was analyzed for the presence of a PDE5 inhibitor using a theoretical calculation.

## 2. Results and Discussion

### 2.1. Structural Characterization

**Figure 1:**
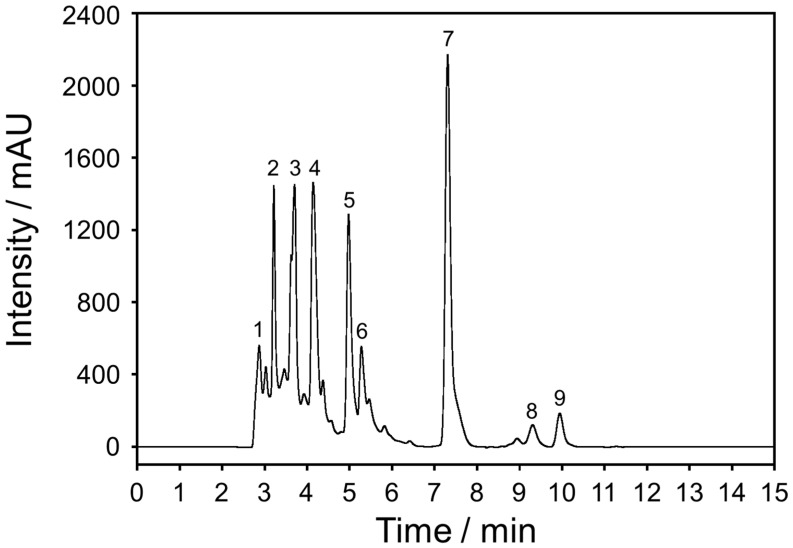
HPLC chromatograms of herbal supplement extract with detection at λ UV absorbance of 226 nm. The spectra profile was obtained by Semi-Prep, ZORBAX SB-C18 column using almost linear isocratic phase of 90% acetonitrile in 100 mM ammonium acetate buffer.

Through the HPLC technique, the extracted solution from an herbal supplement was analyzed and purified into nine fractions, as shown in Figure 1. Among all the fractions, the dominant peak of fraction-7 (F7) was isolated as pale-yellow crystals after recrystallization from dimethylformamide and diethyl ether. Unfortunately, the present work actually focused on structural determination using single crystal X-ray diffraction analysis, however, carrying out routine conventional measurement of the single crystal sample was difficult due to weak crystal structure refinement results. Therefore, the thus-obtained compound has been characterized in terms of its structure by comparing the ^1^H NMR, ^13^C NMR spectroscopy, and mass spectrometry.

**Figure 2:**
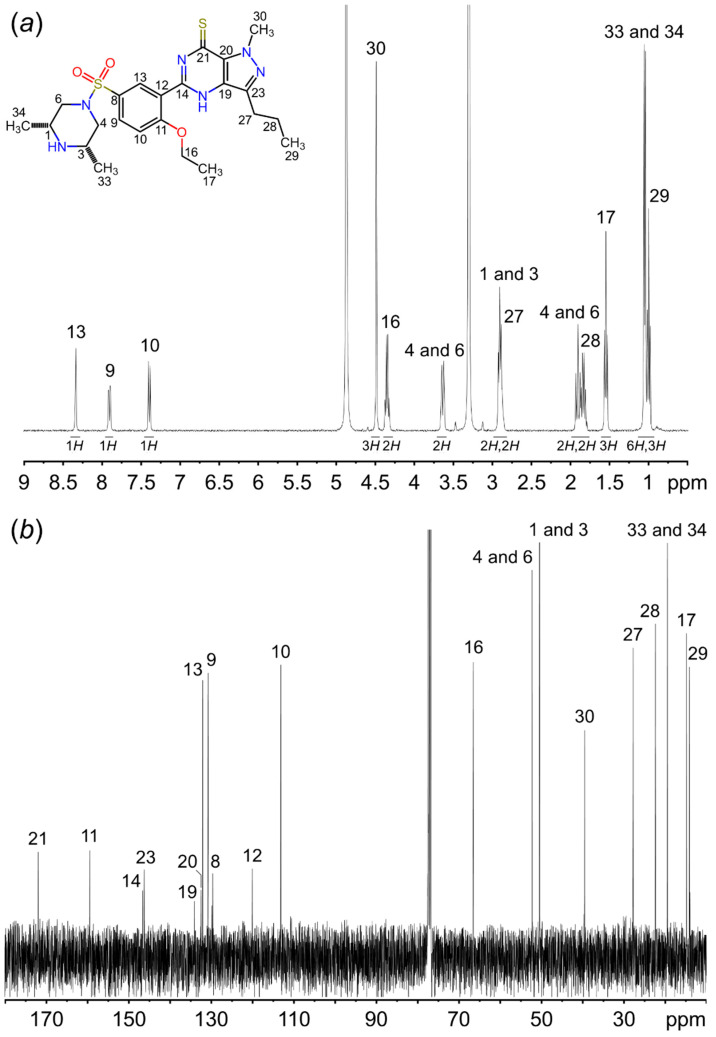
(**a**) ^1^H NMR and (**b**) ^13^C NMR spectrometry of sulfoaildenafil (F7) compound. The spectroscopic numbering used is given in the Figure.

Table 1 shows the NMR spectrometry of F7. The ^1^H and ^13^C NMR spectrums of this compound are shown in Figure 2. In brief, ^1^H NMR spectrum revealed special character of dimethyl piperazine ring at δ_H_ 1.05 (d, *J* = 6.4 Hz, 6H). The methylene protons of piperazine ring signal at δ_H_ 3.64 (d, *J* = 9.4 Hz, 2H) and δ_H_ 1.90 (t, *J* = 10.9 Hz, 2H), which are characterized as the deshielded equatorial protons of a rigid 6-membered ring [1,4,5,12]. The ^13^C NMR spectra indicated five primary carbons; five secondary carbons; five tertiary carbons; and eight quaternary carbons (Table 1). Furthermore, the characteristic structure was confirmed by distortionless enhancement by polarization transfer (DEPT) 90°/135° NMR and ^1^H-^13^C HSQC as shown in Figures S2 and S3 (Supplementary Information).

**Figure 3:**
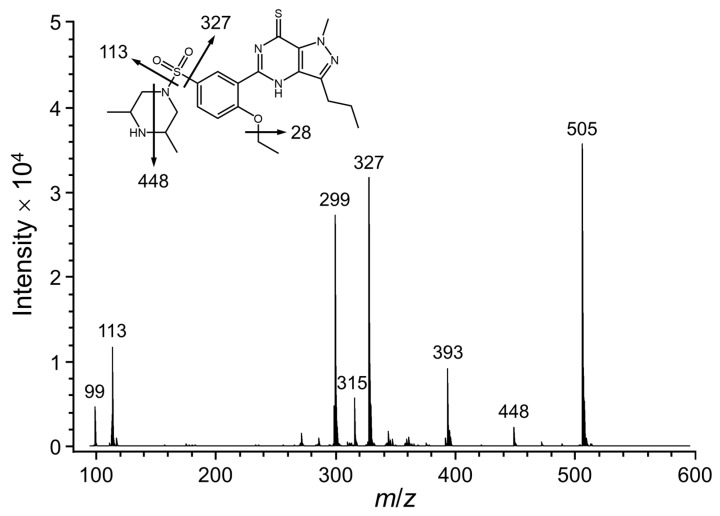
Positive mode MS-MS product ions scan of sulfoaildenafil (F7) at *m*/*z* 505 [M + H]^+^ and fragmentation pathway proposed.

The total ion chromatogram and product ion spectrum for F7 compound are shown in Figure 3. The product ions at *m*/*z* 448, 393, 327, 315, 299, 113, and 99 were observed in the mass spectrometry [1,4,5,6,12]. The fragment ion at *m*/*z* 448 represents a moiety characteristic stemming from the decomposed piperazine ring that contained secondary nitrogen which was observed only for sulfoaildenafil. The compound demonstrates the loss of the piperazine moiety from the molecule, deducing the ion transition from *m*/*z* 505 to 393. The product signal at *m*/*z* 299 was defined by the loss of the ethyl group as a base peak from the fragment at *m*/*z* 327. The molecular ion chromatogram for F7 was also observed at *m*/*z* 505 by ESI-TOF/MS analysis, corresponding to the molecular formula of C_23_H_33_O_3_N_6_S_2_ [M + H]^+^ (Figure S4, Supplementary Information). As the results, the isolated F7 compound was clearly identified as sulfoaildenafil related to the previous studies [4,5,6,12].

### 2.2. Effect of Sulfoaildenafil in Human Umbilical Vein Endothelial Cell Line

The releasing NO triggers vascular endothelial cell through the activity of both inducible nitric oxide synthase (*i*NOS) and endothelial nitric oxide synthase (*e*NOS) enzymes. NO production stimulates the cyclic guanosine monophosphate (cGMP) synthesis via guanylyl cyclase enzyme in endothelial cells, which induce to smooth muscle relaxation, vasodilation, and penile erection, respectively [16]. The feedback loop mechanism of cGMP elevation increases phosphodiesterase type 5 (PDE5) gene expression and enzyme activity which transform into GMP in the smooth muscle, leading to decrease the erection of penile [19]. Sildenafil is an orally active PDE5 inhibitor for the treatment of penile erection dysfunction [20].

**Figure 4:**
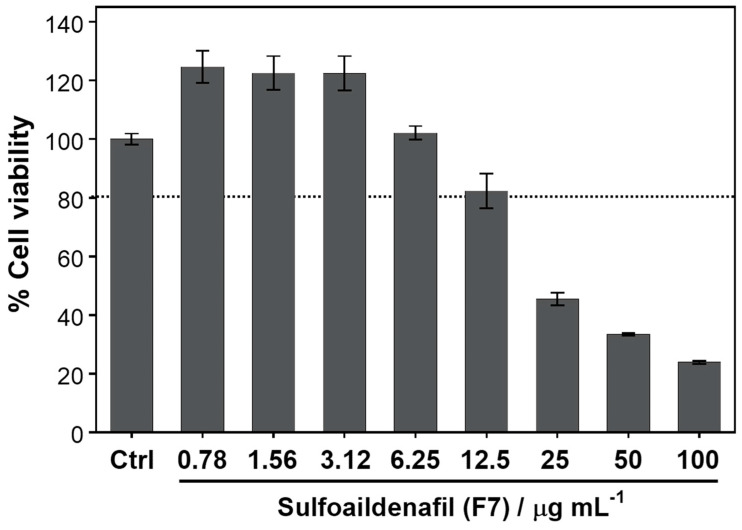
Effects of sulfoaildenafil on cell viability of Ea.hy926 cell by MTT assay at concentrations ranging from 0 to 100 µg mL^−1^ for 24 h. Data are expressed as mean ± SD.

Firstly, the cytotoxicity of the sulfoaildenafil was evaluated that provided more than 80% of Ea.hy926 endothelial cell lines survival rate at the concentration of less than 12.5 µg mL^−1^ as seen in Figure 4. Thus, this compound at the concentration of 10 µg mL^−1^ was chosen to use in further experimental studies.

**Figure 5:**
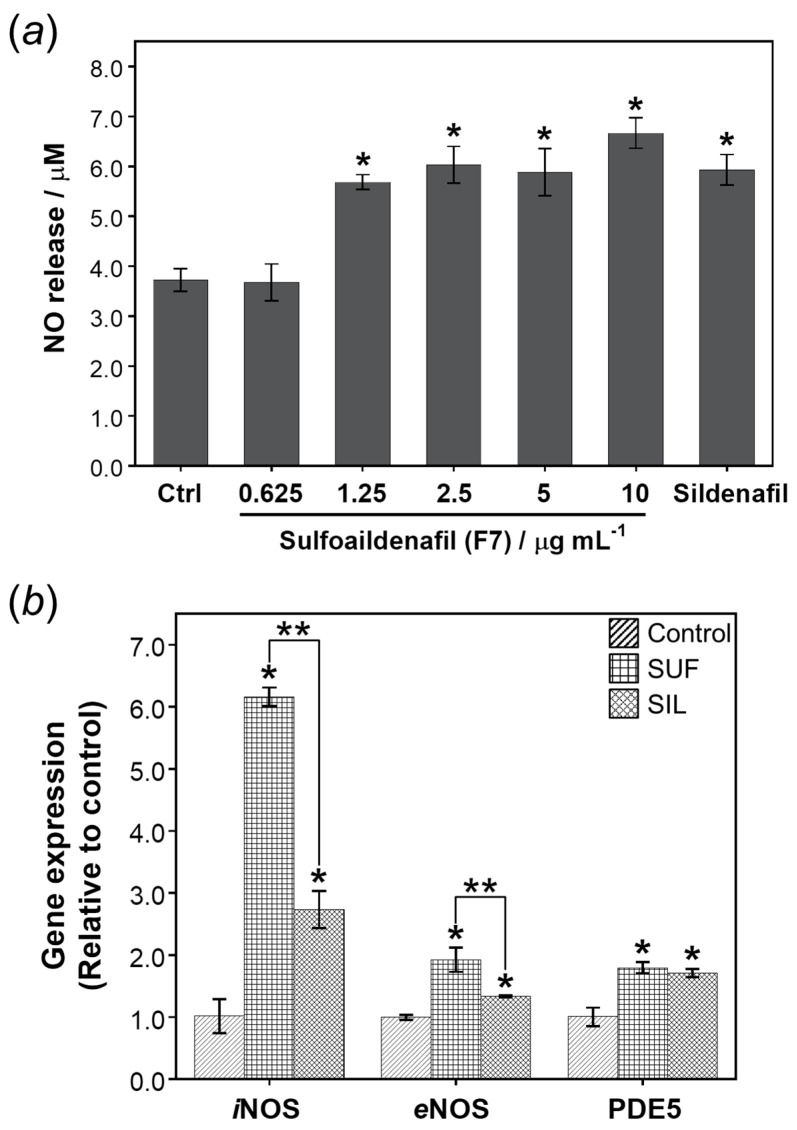
The effect of sulfoaildenafil on (**a**) NO-releasing and (**b**) gene expression of *i*NOS, *e*NOS and PDE5 in Ea.hy926 cell lines. Sildenafil at concentration 10 µg mL^−1^ was the positive control. Bar graphs show mean values ± SD (*n* = 3). Statistical significance was determine using *t*-test at *p* ≤ 0.05 (*) from control. Double asterisks (**) connected with solid lines indicate significance levels of one-sample *t*-test at *p* ≤ 0.05 comparison to sildenafil as positive control.

For NO determination, in Figure 5a, sulfoaildenafil has significantly increased the releasing of NO in the concentration range of 1.25–10 µg mL^−1^ compared to the cell culture medium control (Ctrl). Similarly, sildenafil was found to significantly elevate NO production in endothelial cell lines (Figure 5a). As reported by previous literatures [16,17], the material of sildenafil has been reported to increase the NO releasing in the human umbilical vein endothelial cells in insulin resistance conditions and Ea.hy926 endothelial cell lines.

According to the NO releasing, it is generated by nitric oxide synthase (*i*NOS and *e*NOS) in endothelial cells or triggered endothelial cells by itself or an exogenous source such as NO donor drugs. As expected, sulfoaildenafil, that was able to significantly elevate the NO production (Figure 5a), can up-regulate the levels of *i*NOS and *e*NOS gene expression in Ea.hy926 endothelial cells corresponding to sildenafil [21] used as positive control as showed in Figure 5b. Surprisingly, the active compound of sulfoaildenafil significantly stimulated the upregulation of both *i*NOS and *e*NOS genes at greater levels than that of sildenafil, as illustrated in double asterisks connected with solid lines in Figure 5b. Furthermore, sulfoaildenafil, at the same time, significantly motivated the *PDE5A* gene upregulation as well as sildenafil in comparison with the cell culture medium control [22].

Altogether, these results indicated that sulfoaildenafil, comparing to sildenafil material, enhanced NO production through the *i*NOS and *e*NOS gene expression, which also subsequently up-regulated PDE5 gene expression. This is the first time studying about sulfoaildenafil biological effects, a thioketone analogue of sildenafil, on the erectile dysfunction in the in vitro experimental approach.

### 2.3. Computational Studies

As per the above results, the characteristics of sulfoaildenafil was revealed by using a combination of NMR and mass spectroscopy techniques as well as the biological activities. According to the most well-known PDE5 inhibitor of sildenafil, for this reason, the model compound of sulfoaildenafil, an analog of sildenafil, was used as an active material for molecular docking and molecular dynamics simulation approaches.

#### 2.3.1. Molecular Docking Study

Based on the crystal structure of PDE5, the potential binding activity has been described by subdividing it into three main regions, namely: *(i)* A metal-binding pocket (M pocket), *(ii)* a solvent-filled hydrophilic side pocket (S pocket), and *(iii)* a pocket containing the purine-selective glutamine and hydrophobic clamp (Q pocket) [23]. Here, the molecular docking approach was firstly performed to predict the bioactive binding modes and affinity of the PDE5 inhibitor on the target protein. It should be noted that all models of the well-known PDE5 inhibitors were found to occupy part of the Q pocket (Gln817 and Leu804) at the immediate vicinity of the binding site with the pyrazolopyrimidinone ring of the inhibitors, suggesting the above-mentioned drugs can be accommodated in PDE5 protein and also present PDE5 inhibitor activity [24]. The binding modes were observed at the same site with slightly different binding conformations compared with the sildenafil as a common drug used as a PDE5 inhibitor (Figure S5, Supplementary Information).

Each compound shows favorable binding energy, with such results obtained from AutoDock Vina falling in the range of −10.2~−8.9 kcal mol^−1^. Table 2 shows an observed binding affinity and common amino acid binding residues within 5 Å that was identified to play a key role in the potential activity for PDE5 inhibition (see Table S1, Supplementary Information). Although a lower estimated value of the binding affinity indicates stronger interactions of the protein–ligand complex, the small binding energy difference among these complexes is only 0.5~1 kcal mol^−1^. The interactions of each drug with the potential site of PDE5 were mediated by the hydrophilic/hydrophobic interactions as supported by the findings in previous studies [23,24,25]. As a combined result of experimental study, the subtle differences that were found in the estimated binding energy have led us to further investigate the obtained complex by a comparison between sildenafil and sulfoaildenafil using MD simulations.

#### 2.3.2. Molecular Dynamics Simulations

To enhance the configuration space for sampling accessibility to the molecular geometries, 100 ns long-time simulations of PDE5 with and without the addition of sildenafil and sulfoaildenafil were performed. The structural stability of the proteins as well as the position of the ligands in the binding site cleft were monitored using root mean square deviations (RMSD) with respect to their optimized initial structure (Figure S6, Supplementary Information). Steady oscillation and small fluctuation of RMSD were observed, indicating that the previous complexes were more stable and endured lesser conformational changes during simulations.

##### Binding Free Energy Evaluation

To demonstrate the binding interaction of the complex systems, the values of the relative binding free energy (ΔG*_binding_*) obtained from MM-PBSA protocol were calculated as listed in Table 3. The results showed that the sildenafil (ΔG*_binding_* = −20.34 kcal mol^−1^) slightly binds to the PDE5 protein better than sulfoaildenafil (ΔG*_binding_* = −15.45 kcal mol^−1^) with an energy difference of ~5 kcal mol^−1^. The same tendency of energy values between MM-PBSA and docking calculations were observed.

This slight decrease in the size of the binding free energy of sulfoaildenafil correlated with the shifts of the unfavorable term in (*i*) the van der Waals (vdW) interaction by 10.77 kcal mol^−1^, (ii) the intermolecular electrostatic interactions (EEL) by 41.45 kcal mol^−1^, and (*iii*) the entropy configuration by 2.38 kcal mol^−1^. The change in the contribution from the desolvation of non-polar groups (ENPOLAR) is almost zero. The polar solvation free energy (EPS) of sulfoaildenafil is less unfavorable over 2 times relative to the sildenafil, being shifted by −44.29 kcal mol^−1^.

Nevertheless, this difference in EPS term is not sufficient to compensate for the loss in the vdW causing drug resistance. Unfavorable shifts in EEL and vdW terms of sulfoaildenafil are overcompensated by favorable change in the EPS interaction free energy, leading to an improved affinity in comparison to sildenafil. It can be highlighted that the structural inspection alone may not be sufficient for identifying the key contributions to binding affinity, where the effects of solvation term are taken into account.

**Figure 6:**
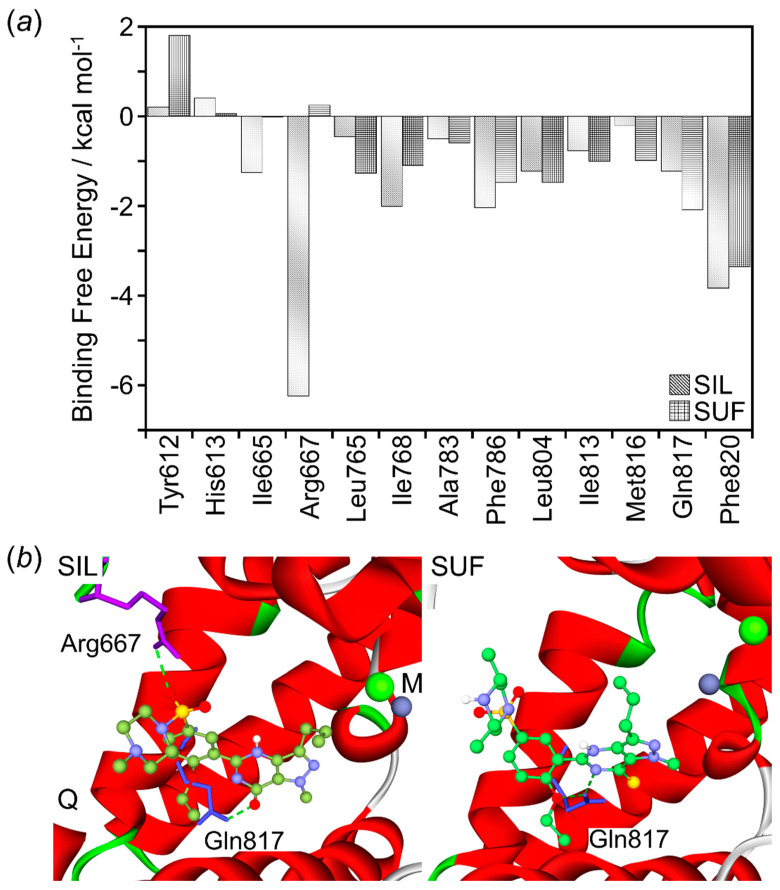
(**a**) Per-residue free energy decomposition of amino acid residues at the binding region of PDE5 in sildenafil and sulfoaildenafil bound systems. All values were given in kcal mol^−1^. (**b**) Final conformations of specificity potential sites with hydrogen bonds formation defined by the binding of sildenafil- and sulfoaildenafil-bound complexes.

The contributions of the essential amino acids to the binding interaction have been investigated by calculating per-residue free energy decomposition. Figure 6a presents the decomposed per-residue free energy upon the binding of each complex system. The negative and positive values represent favorable and unfavorable contributions, respectively.

According to the Figure 6a, the hydrophobic amino acids (Ile665, Ile768, and Phe796) and one electrically charged residue (Arg667) of PDE5 have more favorable interactions with strong binding affinity for sildenafil than that of sulfoaildenafil. On the other hand, the sulfoaildenafil showed more favorable contact with three hydrophobic residues (Leu765, Leu804, and Met816) and one essential neutral-charged amino acid (Gln817) from the active site of PDE5. In addition, we further evaluated the per-residue free energy decomposition of the key binding residue based on the free energy of vdW and the sum of ELE interactions (Figure S7, Supplementary Information). It can be noted that the most favorable contribution of the binding free energy of sulfoaildenafil-bound system was essentially both the vdW and ELE decomposition energies, consisting of Tyr612, Ile813, Met816, Gln817, and Phe820, while the energy term of the vdW was dominant for sildenafil-bound systems including Ile665, Ile768, Phe786, and Leu804. This precisely indicates dynamics interactions upon the binding modes of sildenafil and sulfoaildenafil on the PDE5 protein.

##### Hydrogen Bond Analysis

Analysis of hydrogen bond formation was conducted, and more than a 10% occupancy rate was presented as listed in Table 4. Gln817 of PDE5 was shown to contribute to the key residue interaction with a markedly a high occupancy of hydrogen bonding to interact with both inhibitors. Arg667 of PDE5 that showed favorable binding interactions in the decomposition analysis was found to form hydrogen bonds with sildenafil with a high occupancy rate. On the other hand, sulfoaildenafil is oriented in the potential site through negligible hydrogen bonding with proton-accepting Ser663, while there is no proton acceptor found in the sildenafil.

Figure 6b shows the final conformations of sildenafil- and sulfoaildenafil-bound PDE5 complexes at 100 ns simulations, with hydrogen bond-forming residues shown in stick representation. Using the number of hydrogen bonds between the inhibitors and the potential residues in PDE5 alone is able to explain the reason that the binding free energy is distinctly different from each other.

##### Dynamic Cross-Correlation Matrix (DCCM) Analysis

**Figure 7:**
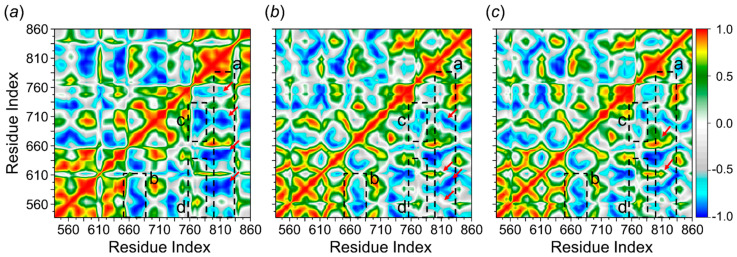
Dynamic cross-correlation diagrams of the fluctuations of Cα–atoms of PDE5 in the complex of (**a**) sildenafil, (**b**) sulfoaildenafil, and (**c**) free PDE5 protein. Positive and negative values are represented in range of color red to royal blue, respectively. The diagonal square relates to the correlation of a residue with itself, i.e., only a region remarked to have highly positive values (red), while the off-diagonal elements represent to an inter-residue correlation (cross-correlations). The binding regions of PDE5, at residues of a, 800~820; b, 640~670, c and d, 760~790, are demarcated with dashed lines.

To observe the conformational changes of PDE5 protein upon the binding effect of sildenafil and sulfoaildenafil, DCCM analysis was conducted to evaluate the occurrence of dynamic motions for residue correlations based on the positions of Cα–atoms of free PDE5 and ligand-bound complex. We perform an initial visual inspection of the dynamic maps obtained from MD simulation period. As illustrated in Figure 7, the diagonal elements of the correlation maps describe fluctuation of individual residues, while the off-diagonal elements represent to an inter-residue correlation (cross-correlations) [27,28]. The cross-correlation coefficients range from a value of −1 (blue-grey regions) to a value of +1 (red to yellow regions). It seems from the correlation map that an overall positive correlation is observed in the case of free PDE5, confirming conformational changes after the ligand binding (Figure 7c).

After ligand binding, DCCM map revealed that both of the ligands effect on the structure conformation of PDE5 protein as illustrated by the change in dynamic patterns and correlations. Firstly, on the Q pocket regions, with residues being 800~820, sildenafil-bound PDE5 was a remarkable decrease in the positive correlated motion (red arrows, in Figure 7a) than that of the sulfoaildenafil-bound complex (red arrows, a in Figure 7b). This result agrees well with the findings of previous studies that found more negative cross-correlation coefficients in the protein part, which arise from an external perturbation of small ligand binding [29,30]. According to the decomposition energy (Figure 6a), sildenafil triggered correlated motion change (blue-grey region) as opposed to sulfoaildenafil in residues around 640~670 (b in Figure 7). The decrease in correlated motions was observed within residues 760~790 for both of ligands, as seen in blue region (c in Figure 7) and grey region (d in Figure 7).

## 3. Materials and Methods

### 3.1. Materials

Sildenafil citrate (Viagra^®^) 100 mg tablets were purchased from Pfizer Labs (Division of Pfizer Inc., NY, NY, USA). Griess’s reagent was obtained from Merck (Sigma-Aldrich Pte Ltd., Singapore). HPLC grade acetonitrile, propa-2-ol, and methanol were purchased from RCI Labscan (RCI Labscan Limited, Bangkok, Thailand). The analytical grade of acetone, methanol, and chloroform solvents were purchased from RCI Labscan (RCI Labscan Limited, Bangkok, Thailand). Formic acid for LC/MS was purchased from Fisher Chemical (Pardubice, Czech Replublic). Ammonium acetate was purchased from Ajax Finechem (Part of Thermo Fisher Scientific, North Ryde, Australia). The 18 MΩ cm deionized water was generated using a Milli-Q system (Millipore, Bedford, MA, USA). Illustra RNAspin Mini RNA Isolation Kit was purchased from Cytiva (Formerly GE Healthcare Life Sciences, Wien, Austria). Tetro cDNA Synthesis Kit and SensiFAST^TM^ SYBR^®^ Lo-ROX Kit were purchased from Bioline (Singapore). The primer was obtained from Invitrogen^TM^. Reagents used in cell culturing were acquired from Gibco (Thermo Fisher Scientific, Life Technologies Corporation, New York, NY, USA). All chemicals used in this study were of analytical grade.

### 3.2. Herbal Supplement Preparations

Four capsules of dietary supplement (600 mg/capsule) were treated with 70% acetonitrile by ultrasonic shaking at room temperature for 60 min before centrifuging. The solid material remaining in the centrifuge tube was repeatedly extracted for 3-times in the same manner. The filtrated-supernatants were recovered and evaporated under reduced pressure using rotary evaporator at 60 °C until dry to obtain the dark-brown color extract (360 mg). The dried-extract sample was stored at 4 °C until next further analysis.

### 3.3. Purification of the Extract Sample by HPLC Analysis

The extracted materials from herbal supplement were isolated by reverse-phase chromatography using high-performance liquid chromatography (HPLC) technique. The freeze-dried materials were dissolved in acetonitrile at room temperature and then filtered through a 0.45 µm Nylon filter (Fisher Scientific, Merelbeke, Belgium). HPLC analysis was performed on an Agilent 1260 Infinity series HPLC equipped with binary pumping system (Agilent Technologies (Thailand) Co. Ltd., Bangkok, Thailand). A Semi-Prep, ZORBAX SB-C18 column (9.4 × 250 mm, 80Å, 5 µm) was used to purify the extract materials. The HPLC condition was performed at a flow rate of 2.5 mL min^−1^, consisted of (A) acetonitrile, and (B) 100 mM ammonium acetate (pH 6.5) buffer. The isocratic mobile phase was used starting with 100% (B) before changing linearly to 10% (B) over 5 min, and holding at 10% (B) for 20 min. The column was re-equilibrated for 5 min prior to the start of the next process. The chromatogram spectra were set at 226 nm. Each high-fractionated peak was collected using Agilent 1260 Infinity fraction collector. The main subsequent fractions of crude extract were collected, and the combined fractionations were recovered. Among all the fractions, the highest mountain peak (F7) was observed as pale-yellow crystals after recrystallization from dimethylformamide and diethyl ether that was used for the structural characterization.

### 3.4. Characterizations

#### 3.4.1. NMR Analysis

Nuclear magnetic resonance (NMR) was measured by a Bruker DPX 400 NMR spectrometer (Bruker UK Limited, Coventry, UK) with a 5 mm multinuclear inverse probe at 296 K. The ^1^H and ^13^C spectra were observed at 400 and 100 MHz, respectively. Crystalline solid of active compound, approximately 6 mg, was dissolved with chloroform-*d* as a solvent for NMR spectroscopy analysis.

#### 3.4.2. ESI-TOF/MS Measurement

The high-resolution mass spectrum was acquired on a MicrOTOF-QII (Bruker Daltonics, Bremen, Germany). The concentration 1.0 µg mL^−1^ of active compound infused directly into the ESI-TOF/MS spectrometer with sodium formate as an internal standard. The measurement conditions for TOF-MS were set as follows: Positive ion electrospray mode, capillary exit voltage at 4.5 kV. The MS data were recorded in the full scan mode in range of *m*/*z* 50–1000.

#### 3.4.3. Ultra-High-Performance Liquid Chromatography-Triple Quadrupole MS Method (UHPLC/MS/MS)

UHPLC/MS/MS analysis was performed with a Dionex Ultimate 3000 (Thermo Fisher Scientific Inc., MA, USA) separation module connected with a MicrOTOF-QII mass spectrometer (Bruker Daltonics, Bremen, Germany). The isolated compound was dissolved in acetonitrile to a concentration of 1.0 µg mL^−1^. The chromatograms were carried out on a Luna^®^ C18(2) (100 × 2.0 mm, 3.0 µm particle size 100Å; phenomenex^®^, Torrance, CA, USA) at 40 °C.

The mobile phases consisted of 5 mM ammonium acetate and 0.1% formic acid (A) and acetonitrile (B). Gradient elution program was set as follows: 10% (B) for 1 min and increased to 40% (B) in 9 min, raised to 75% (B) in 3.5 min, then further increased to 80% (B) in 2.5 min, and held there for 5 min before decreased to 10% (B) in 0.1 min and equilibrated the column for 3.9 min. The flow rate was set at 0.3 mL min^−1^, and the injected volume was 5 µL. The [M + H]^+^ ions were selected as precursor ion, and MS/MS spectra were acquired. The mass spectrometer was performed in the positive ionization mode, and the spray voltage was set at 4.5 kV with collision energy at 40 eV. The nitrogen served both as auxiliary, collision gas, and nebulizer gas with following parameters: Nebulizer gas at 2.0 Bar, dry gas 7.0 L min^−1^, and dry temperature at 240 °C.

### 3.5. Cell Culture and Treatments

Human umbilical vein endothelial cell line; Ea.hy926 (ATCC^®^ number CRL-2922) was cultured in Dulbecco’s Modified Eagle Medium (DMEM) containing with 10% fetal bovine serum, 2% hypoxanthine-aminopterin-thymidine (HAT), 100 U mL^−1^ of penicillin-G sodium and 100 µg mL^−1^ of streptomycin at 37 °C in 5% CO_2_. Phytochemicals at indicated concentrations from MTT assay were used to treat the cells into 96-well plate (10,000 cells/well). 10% DMSO was used as the positive control that indicated the cellular toxicity. For determination of NO production and the gene expression of *i*NOS, *e*NOS, and PDE5A, cells were plated in 6-well plate at a density of 50,000 cells/well. The cells were growth arrested at 80% confluency before being used in the experiments. Sildenafil at a concentration 10 µg mL^−1^ was used as a positive control in the in vitro study. After the treatment period, cell lysates were collected for the determination of gene expression levels, while culture supernatants were collected for the measurement of NO releasing.

#### 3.5.1. Measurement of NO Production

For analysis of NO production from the nitrile accumulation in culture media via Griess reaction assay, 100 µL of treated-media samples or sodium nitrile standards (0–100 µM) were mixed with 100 µL of Griess reagent (1% sulfanilamide, 0.1% *N*-(1-naphtyl) ethylenediamine dihydrochloride in 2.5% H_3_PO_4_ solution). The mixture solution was incubated for 10 min at room temperature and the absorbance was measured at 540 nm using a microplate reader. The concentration of NO in each sample was measured to generate a standard curve [31].

#### 3.5.2. Gene Expression Analysis Using Real-Time Reverse Transcriptase Polymerase Chain Reaction (Real-Time RT PCR)

The total RNA was extracted by using Illustra^TM^ RNAspin Mini RNA Isolation Kit. Five hundred nanograms of total RNA was reverse-transcribed into cDNA using Tetro cDNA Synthesis Kit. Real time PCR was conducted to determine the reaction of denaturation, annealing and extension using SensiFAST^TM^ SYBR^®^ Lo-ROX Kit on 7500 Fast Real-Time PCR system (Applied Biosystems^TM^, Thermo Fisher Scientific, New York, NY, USA). The specific primers are shown in Table S2 (Supplementary Information), which were determined the *i*NOS, *e*NOS and PDE5A gene expression in which β-actin was used as the reference constitutive gene. The data were calculated by using the $2^{-\Delta \Delta C_{T}}$ method [32].

#### 3.5.3. Statistical Analysis

The results were displayed as the mean ± standard deviation (SD) of at the least three independent experiments. The statistical differences compared with an among multiple groups were performed by *t*-test. The value of *p* ≤ 0.05 was considered statistically significant.

### 3.6. Computational Analysis

#### 3.6.1. Protein and Ligand Preparation:

A complex structure of PDE5 protein containing sildenafil (SIL) was obtained from the X-ray crystallography structure of the Protein Data Bank with PDB code of 2H42 [33]. To prepare the structure for docking, the ligand and all water molecules were removed. Charges and non-polar hydrogen atoms were added using the prepare_receptor4.py script from MGLTools 1.5.6 [34].

The three-dimensional (3D) structures of PDE5 inhibitors, vardenafil (VAF), tadalafil (TAF), and sulfoaildenafil (SUF), were obtained from the National Center for Biotechnology Information with PubChem compound summary for CID135400189, CID110635, and CID56841591, respectively (Figure S1, Supplementary Information). The initial structure was followed by short optimization with gradient tolerance of 0.0100 kcal mol^−1^ Å of root mean squared (RMS) using the software of Discovery Studio visualizer 2019 (3DEXPERIENCE Company, Vélizy-Villacoublay, France) [35]. Individual PDB files were prepared for docking using the prepare_ligand4.py script from MGLTools, using only the largest non-bonded fragment present.

#### 3.6.2. Docking Parameters

The software package of AutoDock Vina [36] was performed for all molecular docking simulation study to anchor the PDE5 inhibitors into the active site of the PDE5 protein. In general, the docking parameters were kept to their default values. The total size of the cubic docking box was set to be 60 Å along each dimension (*x*, *y*, and *z*) by the grid point spacing of 0.375 Å. The ligand molecule from the complex PDB ID:2H42 structure was used for the center of the grid box (*x*, *y*, and *z*; 30.790, 119.342, 11.038). Exhaustiveness parameter corresponding to the amount of sampling effort was set to 100 with the energy range of 10 kcal mol^−1^, and the maximum number of poses to report was set to 20 using the built-in clustering analysis with a 2.0 Å cut-off.

#### 3.6.3. Molecular Dynamics Simulations and Binding Free Energy Calculation

All molecular dynamics (MD) simulations were performed by PMEMD.CUDA [37,38] from AMBER 18 suite of programs [39] on NVIDIA Geforce GTX-1070 Ti for speeding up the simulation times. All parameters used in this study were set according to the procedures described in previous work [40]. Briefly, the general AMBER force field (GAFF) parameters were carried out to generate the atomic parameters of each ligand and Gasteiger charge was used to assign the charge parameter for all ligands in MD simulations. Each complex structure under periodic boundary conditions was solvated in a cubic box of TIP3P water molecules extending to 10 Å along each direction from the complex model, and Na^+^ ions were added as neutralizing counterions. The cutoff distance was kept to 12 Å in order to compute the non-bonded interactions. The AMBER ff14SB force field parameters were used to apply the description of the complex characterization. The long-range electrostatic were treated using the particle mesh Ewald (PME) method [41,42]. The SHAKE algorithm and Langevin dynamics were applied to constrain the bonds that involved hydrogen atoms and to control the temperature. The time step of 2 fs was set and the trajectory was recorded every 0.2 ps. The temperature was gradually increased from 0 to 310.15 K over a period of 100 ps of NVT dynamics and followed by 5 ns of NPT equilibration at 310.15 K and 1 atm pressure. Finally, a total 100 ns of the production phase NVT-MD simulation was performed for properties collection. Trajectory analyses (root mean square deviation and fluctuation, dynamic cross-correlation, hydrogen bond) were carried out from the production phase MD using CPPTRAJ module in Amber 18 program [43].

Binding free energy calculation of each simulation complex was performed based on selected MD snapshots using Amber molecular mechanics Poisson–Boltzmann surface area (MM-PBSA) and molecular mechanics Generalized Born surface area (MM-GBSA) protocols [44]. The 2500 snapshots were extracted from the trajectory simulation data. The grid size from the PB calculations in MM-PBSA was 0.5 Å. The values of the interior and exterior dielectric constants in MM-GBSA were set to 1 and 80, respectively. The structural images were presented using DS software.

#### 3.6.4. Dynamic Cross-Correlation Matrix Analysis

Dynamic movements between the Cα–atoms in PDE5 protein over the simulation period were quantified in the term of the dynamic cross-correlation matrix (DCCM). DCCM was analyzed using CPPTRAJ module of the AMBER 18 suites. The cross-correlation matrix elements, *C*_ij_, are defined by [27,30]:

$$C_{ij}=\;\frac{‹\Delta r_{i}\;\Delta r_{j}›}{{\left(‹\Delta r_{i}^{2}›‹\Delta r_{j}^{2}›\right)}^{\frac{1}{2}}}$$

where *i* and *j* represents the position vectors of residue in the structure. The displacement vectors in each residue are represented as $\Delta r_{i}$ and $\Delta r_{j}$. The dynamic diagrams are displayed as a color-coded matrix of Pearson correlation coefficients.

The movement towards the same direction between the residue pairs show a positive value (+1) in the color ranges from light green to deep red; while the movement of opposite direction shows a negative value (−1) in the color range from grey to royal blue. The diagonal square relates to the relationship of a residue with itself, i.e., only region remarked to have highly positive values (red), while off-diagonal elements describe inter-residue correlation (cross-correlations).

## 4. Conclusions

Here, we found a synthetic contaminant in herbal aphrodisiacs purchased at a general drug store. The compound was identified as sulfoaildenafil, a thioketone analog of sildenafil. Analytical techniques, including HPLC, LC-MS/MS spectrometry and NMR spectroscopy, were carried out for the isolation, purification, and characterization of this compound. The sulfoaildenafil, which displays structural similarity to synthetic inhibitors of PDE5, has been illegally added to dietary supplements causing subsequent health risks to consumers. The effects of sulfoaildenafil have been investigated for the first time by means of carrying out experiment and theoretical approaches to postulate premising the selective inhibition of PDE5 activity in comparison with the complex of sildenafil as a commercially controlled drug.

The biological results revealed that sulfoaildenafil can affect the therapeutic level of NO through the upregulation of nitric oxide synthase (*i*NOS and *e*NOS) and PDE5 gene expressions. According to the MD simulations, we suggest that sulfoaildenafil as well as sildenafil could be potent inhibitors of PDE5 protein with specific binding mode and affinity of the key residue interactions. Indeed, considering that the resolved complexes between sildenafil- and sulfoaildenafil-bound PDE5 reveal a clear hydrogen bond formation at Gln817 of PDE5 protein, the small binding free energy difference between these compounds is about 5 kcal mol^−1^.

This report provides fundamental knowledge for the screening of adulterants in herbal drugs and the data in this study can be useful for this particular purpose. These are unique features of the potential activity of PDE5 protein and its inhibitors, sildenafil, and sulfoaildenafil; configurations are key considerations for understanding the modes of actions and predicting the biological activity of PDE5 inhibitors. Furthermore, the experimental data gathered herein with regard to the biological functions of sulfoaildenafil, with a focus on the role of toxicity, NO-releasing levels, and gene expression in the in vitro, have supported these concrete results.

## Figures and Tables

**Table 1:** ^1^H, ^13^C NMR, ^1^H-^13^C HSQC, and DEPT (90° and 135°) spectrometry of sulfoaildenafil compound.

Groups	Position	^1^H (δ_H_)		^13^C/^1^H-^13^C HSQC (δ_C_)	DEPT
Piperazine ring	33 and 34	1.05	(d, *J* = 6.4 Hz, 6H)	19.46	CH_3_
	1 and 3	2.92	(d, *J* = 7.4 Hz, 2H)	50.47	CH
	4 and 6	3.64	(d, *J* = 9.4 Hz, 2H)	52.27	CH_2_
		1.90	(t, *J* = 10.9 Hz, 2H)		
Ethoxy group	16	4.35	(q, *J* = 6.8 Hz, 2H)	66.52	CH_2_
(CH_3_CH_2_O)	17	1.55	(t, *J* = 6.9 Hz, 3H)	14.84	CH_3_
Propyl group	27	2.89	(t, 2H)	27.78	CH_2_
(CH_3_CH_2_CH_2_)	28	1.83	(dt, *J* = 14.6, 7.4 Hz, 2H)	22.38	CH_2_
	29	1.00	(t, *J* = 7.4 Hz, 3H)	14.16	CH_3_
Methylamine moiety	30	4.49	(s, 3H)	39.54	CH_3_
(CH_3_N)					
Quaternary carbon	8			129.68	
	11			159.50	
	12			120.08	
	14			146.67	
	19			134.12	
	20			132.51	
	21			171.99	
	23			146.28	
Aromatic ring	10	7.40	(d, ^1^H)	113.18	CH
	9	7.91	(d, ^1^H)	130.82	CH
	13	8.34	(s, ^1^H)	132.11	CH

**Table 2:** Binding affinity (kcal mol^−1^) and common amino acid binding residue in each system by AutoDock Vina.

Models	Binding Affinity	Common Amino Acid Binding Residue within 5 Å
Sildenafil	−9.6	Tyr612, His613, Asn661, Ala767, Val782, Phe786, Leu804, Ile813, Gln817, Phe820
Vardenafil	−8.9	Tyr612, Ser663, Ile665, Leu725, Ala779, Val782, Phe786, Leu804, Ile813, Met816, Gln817, Phe820
Tadalafil	−10.2	Tyr612, Ser663, Ala767, Ala779, Val782, Ala783, Phe786, Leu804, Ile813, Met816, Gln817, Phe820
Sulfoaildenafil	−9.1	Tyr612, His613, Asn662, Ser663, Leu725, Leu765, Ala767, Val782, Ala783, Phe786, Leu804, Ile813, Met816, Ile824, Gln817, Phe820

**Table 3:** Free energy terms (kcal mol^−1^) for PDE5 binding to inhibitors sildenafil and sulfoaildenafil estimated by MM-PBSA method.

Parameter	Sildenafil–PDE5	Sulfoaildenafil–PDE5
ΔG*_binding_*	−20.34 ± 3.54	−15.45 ± 2.12
ΔS	−27.13 ± 2.17	−24.74 ± 1.56
vdW	−65.01 ± 3.01	−54.24 ± 2.17
EEL	−53.04 ± 3.73	−11.59 ± 3.29
EPS	76.35 ± 3.71	32.06 ± 2.52
ENPOLAR	−5.81 ± 0.11	−6.42 ± 0.15

Note: The EEL and vdW represent the electrostatic and van der Waals contributions from MM, respectively. EPS stands for PB electrostatic contribution to the polar solvation free energy, while ENPOLAR is the nonpolar contribution to the solvation free energy. ΔS (kcal mol^−1^, at 298.15 K) is an entropically unfavorable protein-ligand complex energy calculated by normal mode analysis. ΔG*_binding_* (kcal mol^−1^) is the final estimated binding free energy calculated from the terms above (ΔG*_binding_* = ΔE_MM_ + ΔE*_solvation_* − TΔS) [26].

**Table 4:** Hydrogen bond analysis of sildenafil and sulfoaildenafil bound PDE5 protein during the MD simulations.

Complex	Acceptor	Donor	%Occupied	Average Distance_A–D_/Å
Sildenafil	SIL@O	Gln817@NE2	54.85	3.13
(SIL)	SIL@O	Arg667@NH1	43.60	2.86
	SIL@O	Arg667@NH2	40.26	2.88
	SIL@O	Arg667@NH2	14.18	3.16
Sulfoaildenafil	SUF@O	Gln817@NE2	42.53	3.24
(SUF)	SUF@N	Gln817@NE2	11.05	3.08
	SUF@N	Ser129@O	6.34	2.86
	Ser663@O	SUF@N	3.18	3.22

Average distance_A–D_ = average distance between acceptor and donor of heavy atoms.

## Supplementary Materials

The following are available online, Table S1: 2D diagrams in each PDE5-ligand complex surrounding amino acid binding residues with hydrogen bond interaction by molecular docking analysis, Table S2: Oligonucleotide sequences used as primers to amplify iNOS, eNOS, PDE5, and β-Actin by RT-PCR, Figure S1: Chemical structures of the herbal medicals: (a) sildenafil, (b) vadenafil, (c) tadalafil, and (d) sulfoaildenafil, Figure S2: ^13^C NMR spectrometry of (a) DEPT 90° and (b) DEPT 135° of sulfoaildenafil (F7) compound, Figure S3: ^1^H-^13^C HSQC NMR spectra of sulfoaildenafil (F7) compound, Figure S4: Full scan ESI-TOF/MS spectrum for sulfoaildenafil (F7) compound at m/z 505 [M + H]+, Figure S5: Docking models of sildenafil (red), vardenafil (green), tadalafil (cyan), and sulfoaildenafil (black) in binding region of PDE5 protein. The potential site of PDE5 is divided into three pockets; the metal binding pocket (M) shown in pink, the purine-selective glutamine and hydrophobic pocket (Q) shown in blue, and the solvent-filled side pocket (S) shown in green, Figure S6: Root mean square deviations (RMSD) of all Cα-atoms position with respect to their optimized initial structure in sildenafil-, and sulfoaildenafil-bound systems, and free PDE5 model over 100-ns simulation period, Figure S7: Per residue free energy decomposition of the key residues at the potential site of the PDE5 protein. The van der Waals energy (vdW), the sum of the electrostatic (ELE) interactions of the solvation free energy for key residues at the bind site of the PDE5 protein. All values were given in kcal mol^−1^.

## Sample Availability

Samples of the compounds, sildenafil and sulfoaildenafil, are available from the authors.
