# Supplementary material for: Structural Determination, Biological Function, and Molecular Modelling Studies of Sulfoaildenafil Adulterated in Herbal Dietary Supplement
Source: Molecules. 2021 Feb 11;26(4):949. doi: 10.3390/molecules26040949 (PMC7916901; doi:10.3390/molecules26040949)
Supplement: Supplementary file 1 [file molecules-26-00949-s001.pdf]

Supplementary Information

Table S1. 2D diagrams in each PDE5-ligand complex surrounding amino acid binding residues with hydrogen bond interaction by molecular docking analysis.

Models	Amino Acid Binding Pocket with Hydrogen Bonding
**Sildenafil**	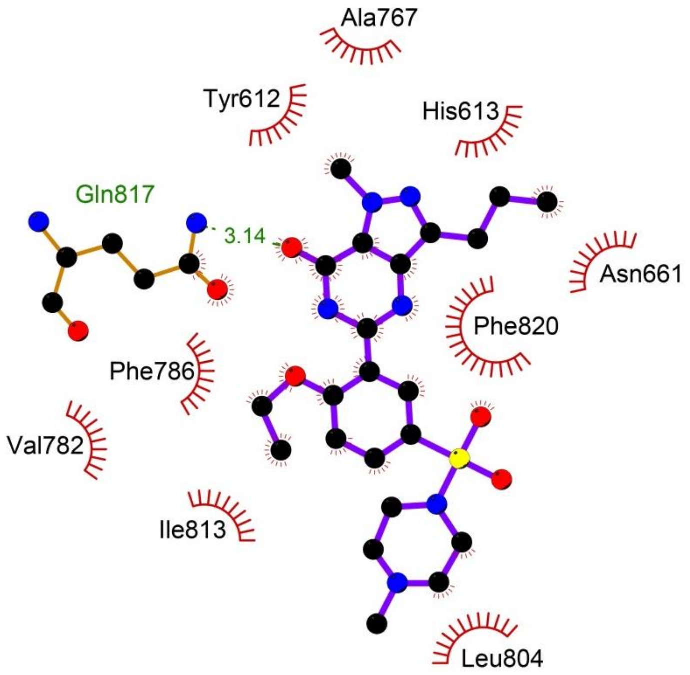
**Vardenafil**	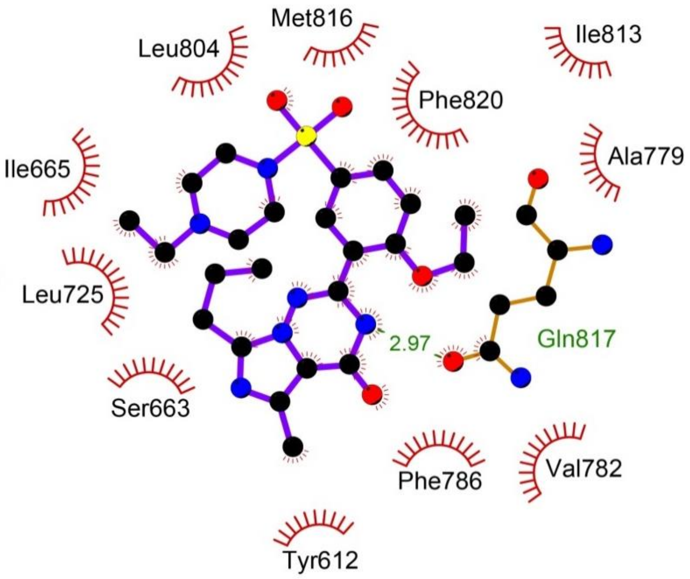

Tadalafil

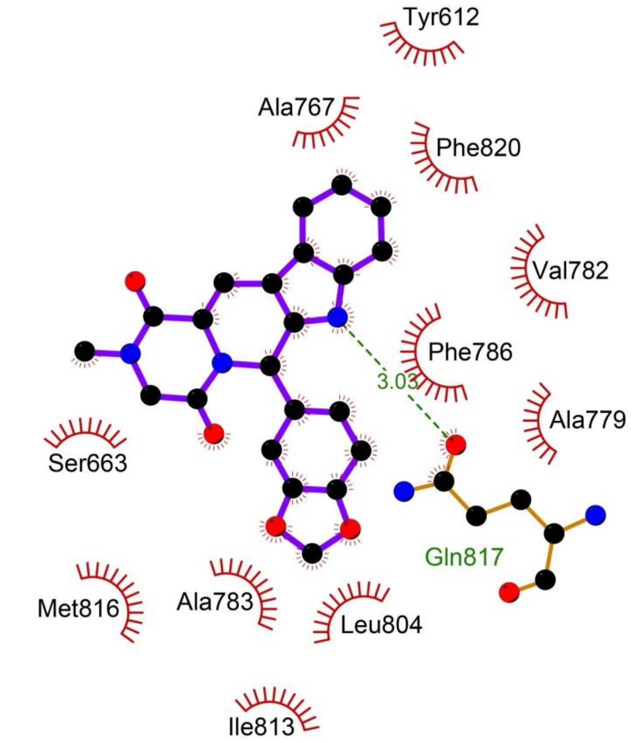

Sulfoaildenafil

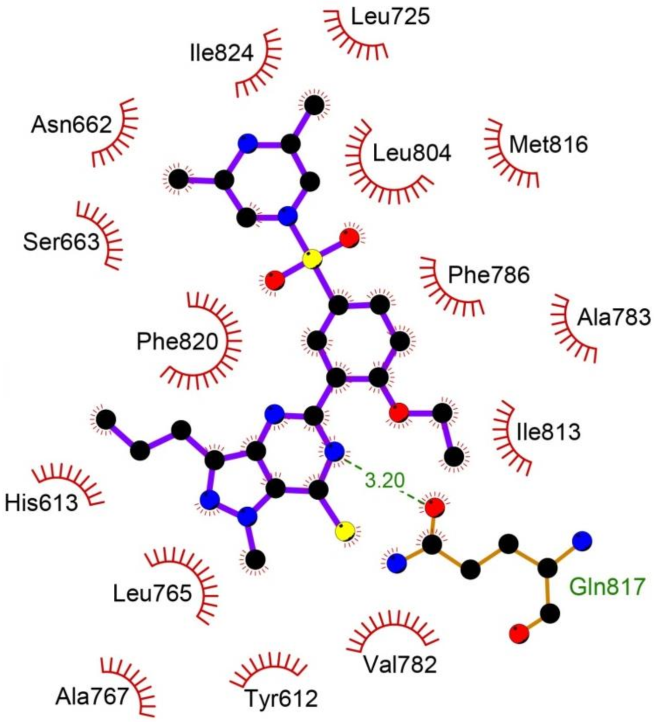

Table S2. Oligonucleotide sequences used as primers to amplify *i*NOS, *e*NOS, PDE5, and β-Actin by RT-PCR.

Genes	Direction	Primer Sequence (5′–3′)	Reference Sequence
*i*NOS	Forward	CTGCTTGAGGTGGGCGG	NM_000625.4
	Reverse	GTGACTCTGACTCGGGACGCC	
*e*NOS	Forward	TGGACCTGGATACCCGGAC	NM_001160109.2
	Reverse	TGGTGACTTTGGCTAGCTGG	
PDE5A	Forward	GAAAAGGACTTTGCTGCTT	NM_033430.3
	Reverse	TGATTTTGTTTGCATCATGT	
Actin beta	Forward	CGTGAAAAGATGACCCAGATCA	NM_001101.5
(ACTB)	Reverse	CACAGCCTGGATGGCTACGT	

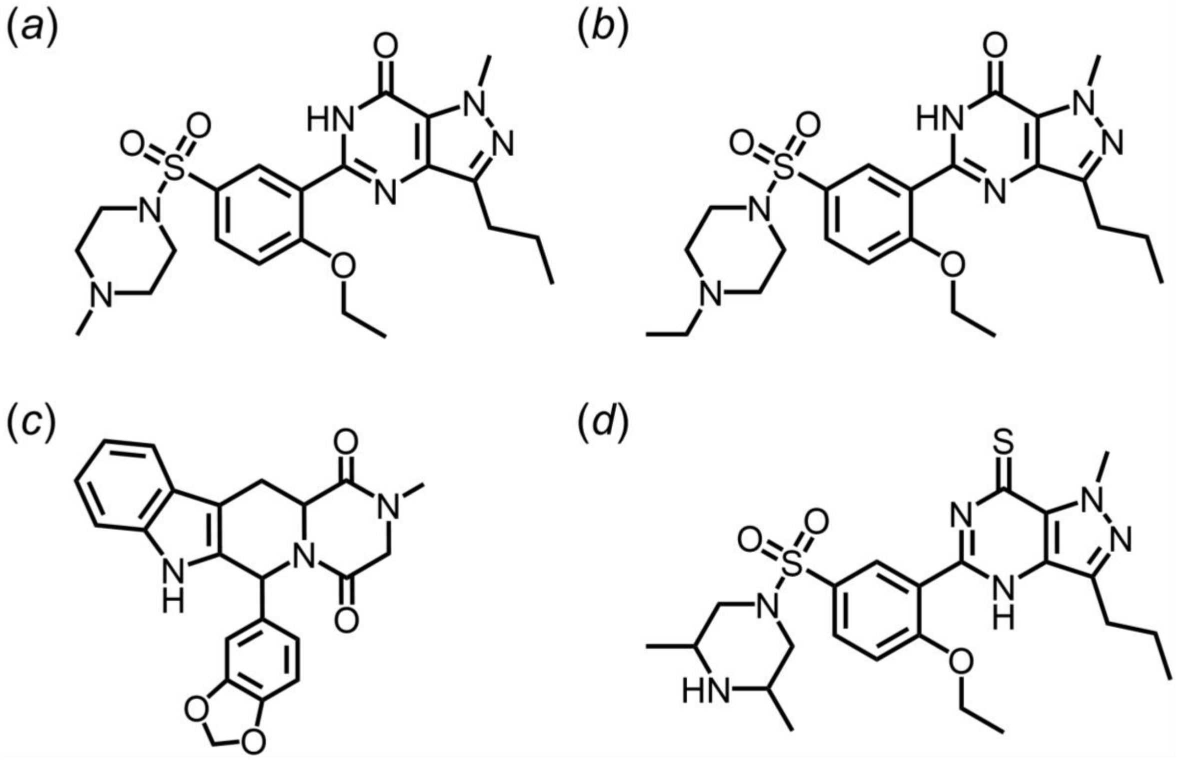

Figure S1. Chemical structures of the herbal medicals: (a) Sildenafil, (b) Vadenafil, (c) Tadalafil, and (d) Sulfoaildenafil.

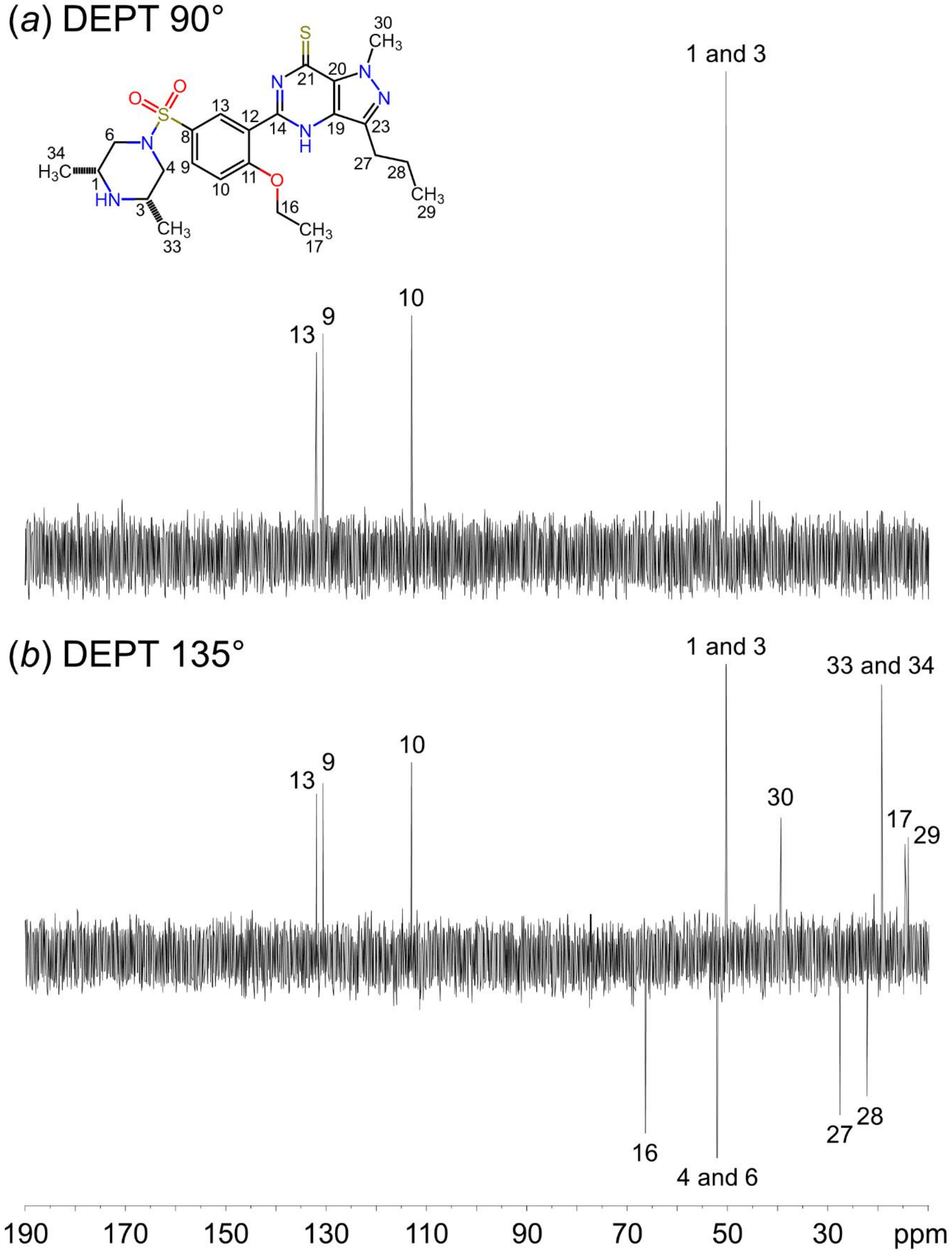

Figure S2. ^{13}C NMR spectrometry of (a) DEPT 90º and (b) DEPT 135º of sulfoaildenafil (F7) compound.

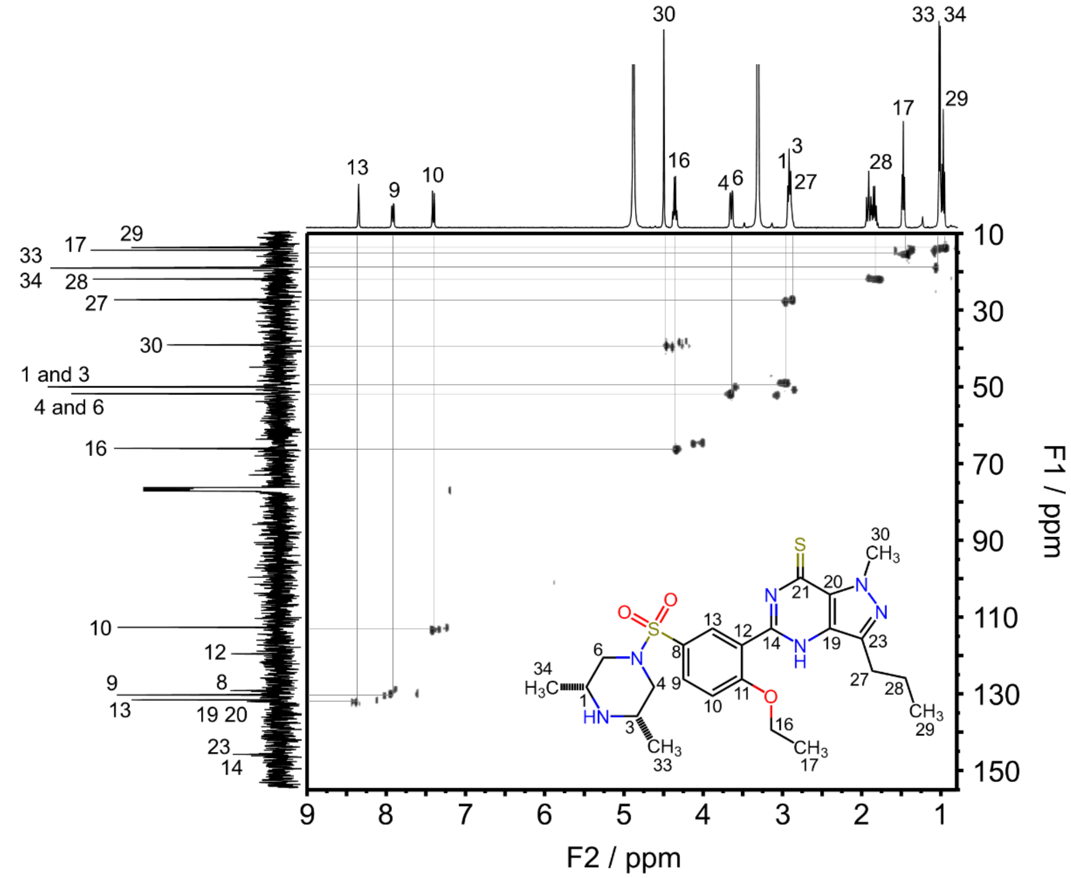

Figure S3. ^{1}H-^{13}C HSQC NMR spectra of sulfoaildenafil (F7) compound.

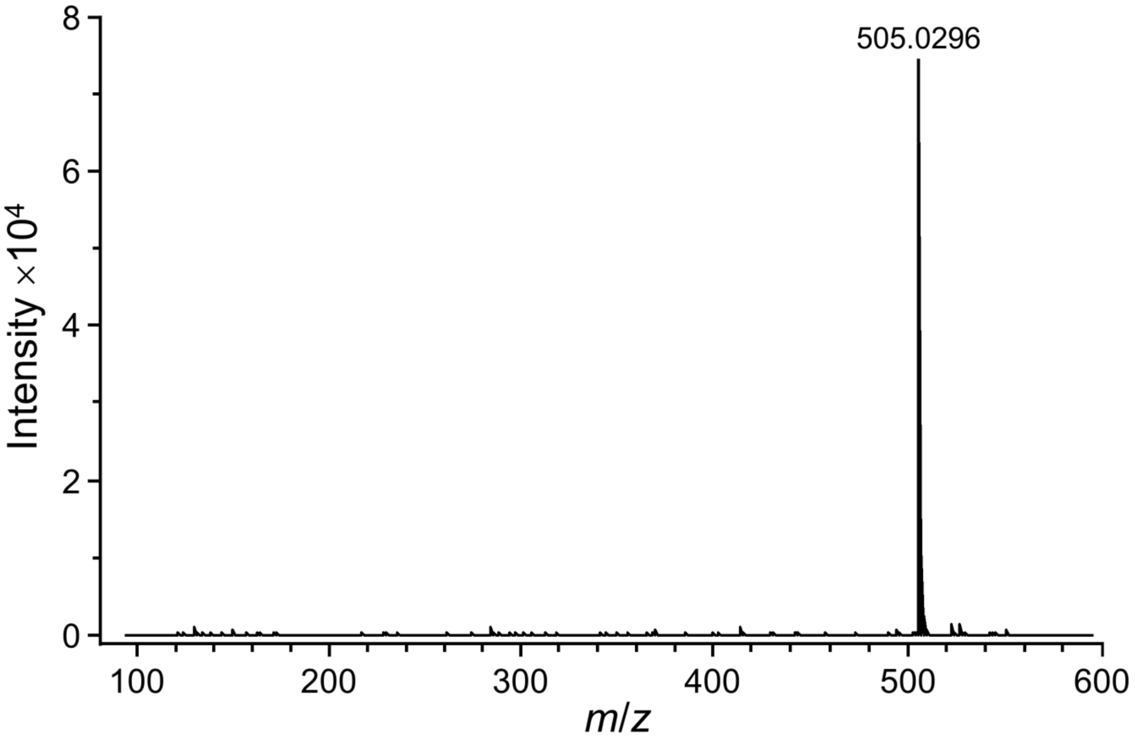

Figure S4. Full scan ESI-TOF/MS spectrum for sulfoaildenafil (F7) compound at *m*/*z* 505 [M + H]$^+$.

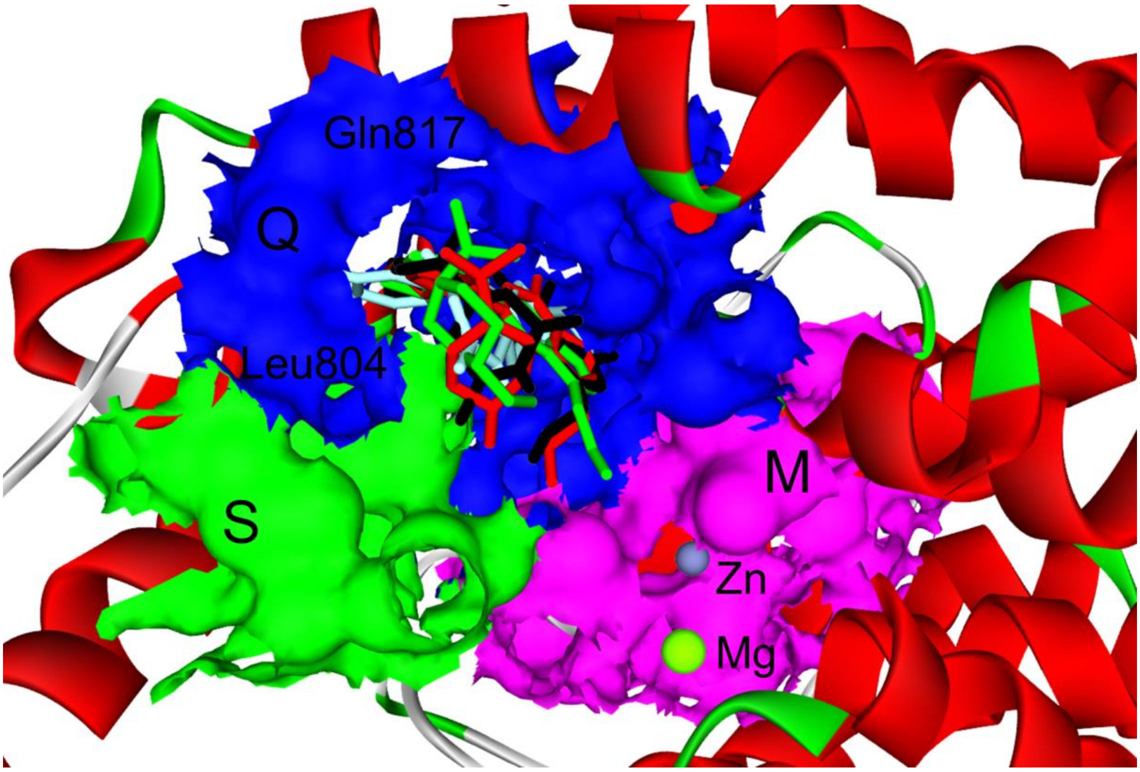

Figure S5. Docking models of sildenafil (red), vardenafil (green), tadalafil (cyan), and sulfoaildenafil (black) in binding region of PDE5 protein. The potential site of PDE5 is divided into three pockets; the metal binding pocket (M) shown in pink, the purine-selective glutamine and hydrophobic pocket (Q) shown in blue, and the solvent-filled side pocket (S) shown in green.

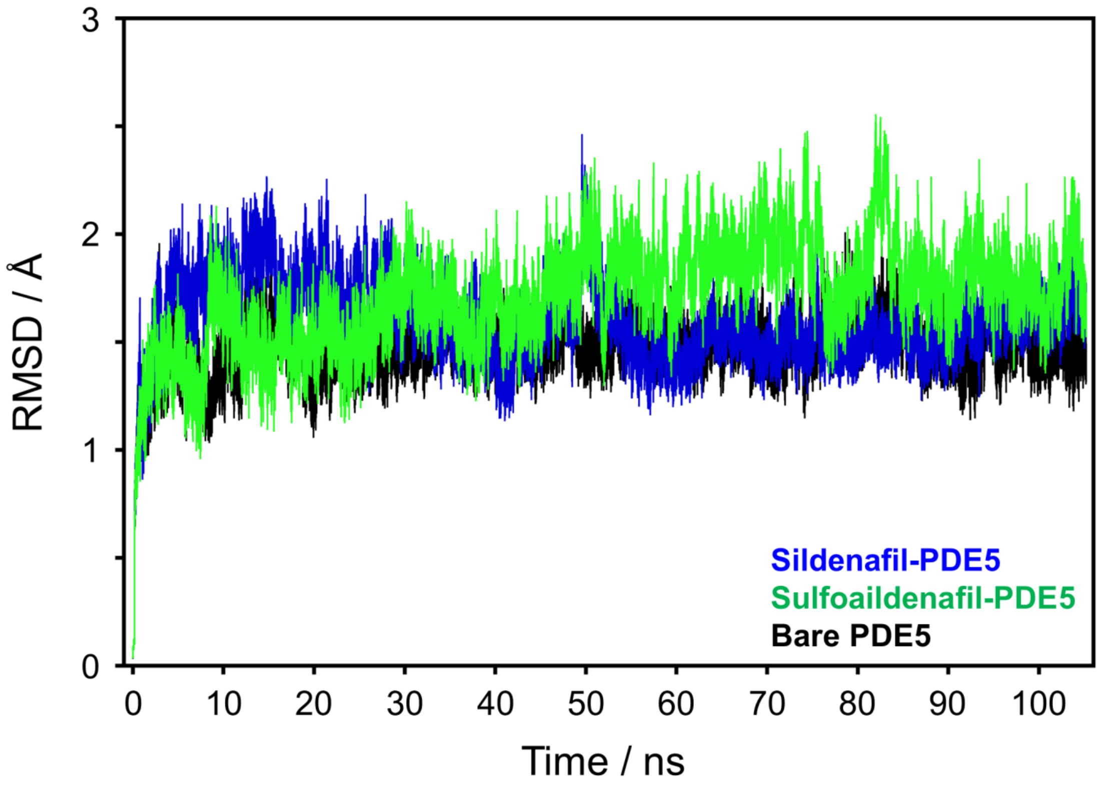

Figure S6. Root mean square deviations (RMSD) of all Cα-atoms position with respect to their optimized initial structure in sildenafil-, and sulfoaildenafil-bound systems, and free PDE5 model over 100-ns MD simulation period.

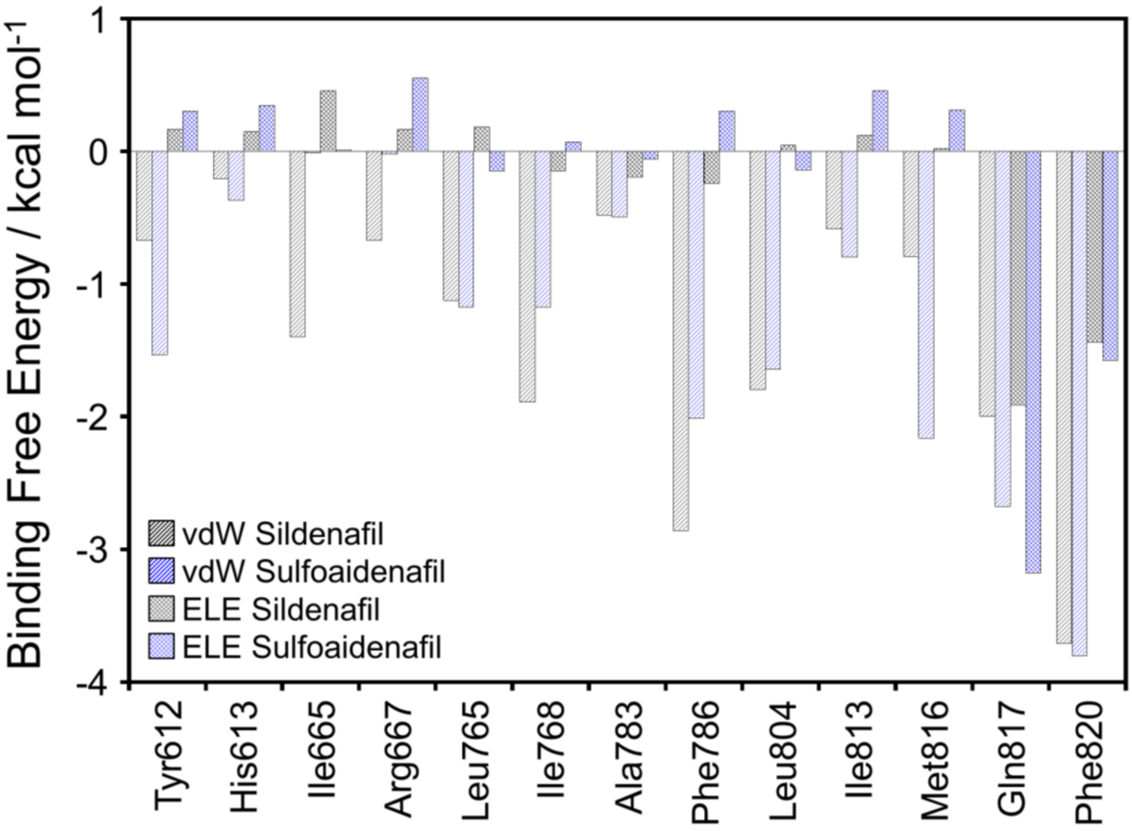

Figure S7. Per residue free energy decomposition of the key residues at the potential site of the PDE5 protein. The van der Waals energy (vdW), the sum of the electrostatic (ELE) interactions of the solvation free energy for key residues at the bind site of the PDE5 protein. All values were given in kcal mol^{-1}.
